# Growing single crystals of two-dimensional covalent organic frameworks enabled by intermediate tracing study

**DOI:** 10.1038/s41467-022-29086-x

**Published:** 2022-03-16

**Authors:** Chengjun Kang, Kuiwei Yang, Zhaoqiang Zhang, Adam K. Usadi, David C. Calabro, Lisa Saunders Baugh, Yuxiang Wang, Jianwen Jiang, Xiaodong Zou, Zhehao Huang, Dan Zhao

**Affiliations:** 1 Department of Chemical and Biomolecular Engineering, National University of Singapore, Singapore, 117585 Singapore; 2 ExxonMobil Asia Pacific Pte. Ltd., 1 HarbourFront Place HarbourFront Tower 1, Singapore, 098633 Singapore; 3 Corporate Strategic Research Laboratory, ExxonMobil Research and Engineering Company, 1545 Route 22 East, Annandale, New Jersey 08801 United States; 4 Department of Materials and Environmental Chemistry, Stockholm University, Stockholm, 10691 Sweden

**Keywords:** Polymer synthesis, Characterization and analytical techniques, Polymers

## Abstract

Resolving single-crystal structures of two-dimensional covalent organic frameworks (2D COFs) is a great challenge, hindered in part by limited strategies for growing high-quality crystals. A better understanding of the growth mechanism facilitates development of methods to grow high-quality 2D COF single crystals. Here, we take a different perspective to explore the 2D COF growth process by tracing growth intermediates. We discover two different growth mechanisms, nucleation and self-healing, in which self-assembly and pre-arrangement of monomers and oligomers are important factors for obtaining highly crystalline 2D COFs. These findings enable us to grow micron-sized 2D single crystalline COF Py-1P. The crystal structure of Py-1P is successfully characterized by three-dimensional electron diffraction (3DED), which confirms that Py-1P does, in part, adopt the widely predicted AA stacking structure. In addition, we find the majority of Py-1P crystals (>90%) have a previously unknown structure, containing 6 stacking layers within one unit cell.

## Abstract (web summary)

Resolving single-crystal structures of two-dimensional covalent organic frameworks (2D COFs) is a great challenge. Here, the authors identify two different growth mechanisms of COFs, enabling the growth and structure determination of micron-sized 2D single-crystalline COFs.

## Introduction

2D COFs are crystalline, porous materials that integrate different organic building blocks into ordered structures via reticular chemistry^1,2^. Given their advantages of high porosity, high stability, and structural tunability, 2D COFs have demonstrated great potential in various applications^3–9^. Despite numerous studies in the 2D COF field since 2005^10^, all the reported 2D COF structures have been characterized through refining powder X-ray diffraction (PXRD) patterns assisted with computational simulations, while direct structural evidence based on single crystals is lacking due to the challenge of growing 2D COF single-crystals^11,12^. Even though there are few reports on the growth of 2D COF single-crystals^13^, their crystal structures are not resolved experimentally. The difficulty for growing 2D COF single crystals with high enough quality for structure resolving originates from their layered structures. Covalent bonds connect the repeating units within every single COF layer; in contrast, adjacent COF layers are held together via relatively weak molecular attractions^14,15^. This structural anisotropy means that the growth of 2D COF crystals requires the coordination of very different forces, including strong chemical bonds and weak molecular interactions. While classical nucleation theory could provide general guidance^16^, crystal growth theory dedicated to 2D COFs is only in its infancy.

Although many 2D COFs have been reported, surprisingly little is known about the molecular-scale mechanism of their formation in solution. Dichtel et al. studied the growth of boronate COF-5 from initially homogenous conditions^17,18^, and proposed different nucleation growth pathways from kinetic Monto Carlo (KMC) simulation^19^. By using transient adsorption spectroscopy, they measured the polymerization speed of the dispersed COF-5 nanoparticles, achieving seeded growth of highly crystalline crystals with diameters as large as 1.5 µm^20^. Clancy et al. examined the growth mechanism of COF-5 by molecular dynamics simulation and quantum mechanical calculations. They investigated the boronate ester bond formation and suggested that COF-5 may grow via template polymerization^21^.

In contrast, our knowledge of growth mechanisms of Schiff-base COFs is even less developed. It is important to understand such growth mechanisms because this class of COFs is one of the most widely studied with the largest variety of molecular precursors^22^. However, only a few studies have been reported on optimizing experimental conditions to improve the crystallinity of Schiff-base COFs^23,24^. Very recently, Dichtel et al. observed that the crystalline structure of imine-linked 2D COFs can be obtained in 60 s, indicating the fast formation kinetics of the crystalline domains^25^.

The above studies contribute to the understanding of 2D COF growth mechanisms. However, critical questions remain unresolved. Taking Schiff-base COFs as an example, why are some easier to crystallize than others? Why is the crystallinity of COFs significantly influenced by the choice of solvents in which they are grown? In the present work, we take a different perspective to explore the growth process of Schiff-base COFs by tracing the intermediates that exist between monomers and COF products. The chemical structures of intermediates can be determined by precisely measuring their molecular weights with mass spectroscopy (MS)^26,27^, then comparing this value with the molecular weight of intermediates predicted from different combinations of monomers. It should be pointed out that MS only characterizes these growth intermediates that are soluble in solution, while oligomers with high molecular weight and insoluble in growth solution cannot be detected by MS. The characterization of intermediates soluble in growth solution can help elucidate scenarios of the COF growth process. In particular, self-assembly and template growth are identified as critical factors for obtaining highly crystalline COFs during the nucleation and self-healing growth stages. These criteria help us select monomers that show promise for the preparation of high-quality single crystals under well-designed conditions. Finally, we present the growth of micron-sized 2D COF single crystals, whose structure is successfully determined by 3D electron diffraction (3DED).

## Results

### Intermediate tracing study on nucleation growth

**Fig. 1:**
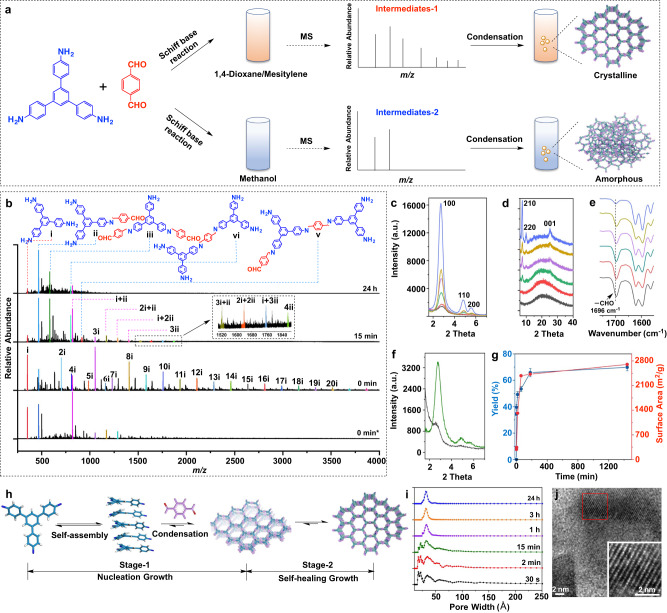
Intermediate tracing study on the growth of TAPB-TA COF. **a** Scheme showing the molecular weight measurement of intermediate species in COF growth solutions by MS. **b** MS spectra of TAPB-TA growth solution using methanol versus 1,4-dioxane/mesitylene mixture as the solvent; *MS spectra taken from methanol growth solution. Quantitative PXRD patterns of the TAPB-TA with different growth time, 2θ value ranging from (**c**) 1.8° to 7.0°, and (**d**) 7.0° to 40°; growth solvent: 1,4-dioxane/mesitylene; growth time: 30 s (black), 2 min (red), 15 min (green), 1 h (purple), 3 h (orange), and 1 day (blue). **e** FTIR spectra of TAPB-TA with growth time of 30 s (black), 2 min (red), 15 min (green), 1 h (purple), 3 h (orange), and 1 day (blue). **f** Quantitative PXRD patterns of TAPB-TA with 15 min growth time in methanol (black) versus 1,4-dioxane/mesitylene (green). **g** Yield and surface area of the TAPB-TA products versus growth time. **h** Scheme showing the two stages that exist in the growth of TAPB-TA COF. **i** Pore size distribution of TAPB-TA products with different growth time. **j** TEM image of the TAPB-TA grown in 30 s.

Herein, we use the 2D COF 1,3,5-tris(4-aminophenyl)benzene/terephthaladehyde (TAPB-TA)^23^ as an example to study its growth mechanism. TAPB-TA was selected because it has one of the simplest, but highly representative, structures in the Schiff-base COF category. The intermediate tracing approach for studying the TAPB-TA growth process is shown in Fig. 1a. Specifically, TAPB and TA were dissolved in 1,4-dioxane/mesitylene (4/1, v/v) to form a clear solution, then the acetic acid aqueous solution was added. The mixture was allowed to react for the different time at 65 °C, the solid COF products were quickly removed from the mixture by filtration, and the clear growth solution was then subjected to MS measurement. For comparison, methanol was also used as a solvent for TAPB-TA growth. As soon as TAPB and TA were dissolved in 1,4-dioxane/mesitylene to form a clear solution, a considerable number of MS signals, ranging from 352 to 7000 g/mol, were detectable (Fig. 1b). The observation of compound-ii (Fig. 1b) indicates the beginning of polycondensation between TAPB and TA. At this point, no solid COF product was obtained. The majority of MS signals have molecular weight exactly 1 to 20 times of the TAPB (Fig. 1b, Supplementary Fig. 1, Supplementary Table 1). This is an intriguing observation, implying the aggregation of TAPB in the growth solution. In contrast, when methanol was used as the solvent, only a few MS signals were observed, indicating much less TAPB aggregation in methanol.

As the reaction proceeded to 30 s, light yellowish COF powders were obtained. The yield was as high as 32%, indicating a fast polycondensation rate. PXRD analysis indicated a clear signal arising from [100] reflection with a 2θ value of 2.7° (Fig. 1c). The observation of crystalline COF domains was further supported by transmission electron microscopy (TEM) images of crystalline lattices, which show a *d*_001_ = 3.5 Å (Fig. 1j). Such rapid crystallization of TAPB-TA agrees well with the previous report^25^. Fourier-transform infrared (FTIR) shows that a significant amount of unreacted aldehyde groups remains in the COF product (Fig. 1e), suggesting that the obtained COF has many defects with a low polymerization degree. Accordingly, intermediates of compound-iii to -v (Fig. 1b) were detected in the growth solution. These intermediate species are too small to be nanosheets, but likely represent products from intermediate stages of condensation producing oligomers that feed the formation of the nanosheets. This observation contradicts the suggested mechanism that only monomers polymerize into nanosheets first and then stack together to form crystalline COFs. Notably, after 30 s of polymerization, most MS signals having integer multiple masses of TAPB disappeared (Supplementary Fig. 2, 2i to 20i), while MS signals from the aggregates of oligomers (i+ii to 4ii) were observed, implying that TAPB aggregates reacted with TA to form new aggregates. Because some of the new aggregates were too big to be stable in the solution, they precipitated as COF products. Meanwhile, others were small enough to remain in the growth solution.

**Fig. 2:**
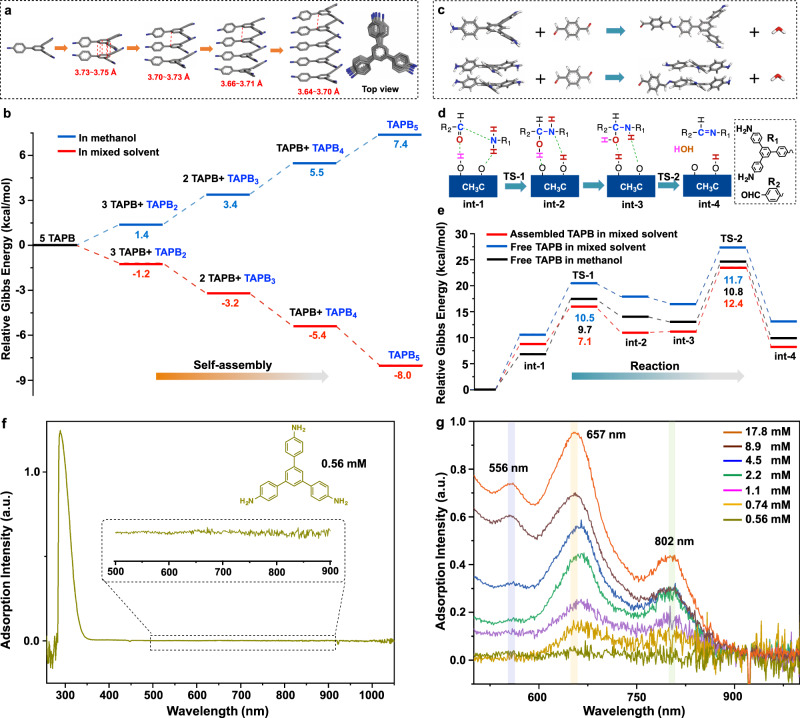
Self-assembly and Schiff-base condensation of TAPB monomer. DFT calculations on the Self-assembly and Schiff-base condensation of TAPB monomer: **a** DFT-calculated assembly structures of TAPB. **b** Gibbs energy variation during the self-assembly of TAPB in methanol versus 1,4-dioxane/mesitylene. **c** Reactions between TAPB and its stacked structure with TA. **d** Proposed Schiff-base formation mechanism between TAPB and TA. **e** Gibbs energy variation during the reactions of TAPB and its assembled structures with TA in methanol versus 1,4-dioxane/mesitylene. **f** UV-Vis spectrum of TAPB with a concentration of 0.56 mM in TAPB-TA COF growth solution. **g** UV-Vis spectra of TAPB with different concentrations in TAPB-TA COF growth solution. The UV-Vis adsorption intensity of each spectrum was normalized according to concentration.

Density functional theory (DFT) calculations were applied to further understand the growth process. The calculation results suggest that TAPB self-assembles into stacked structures during COF growth. Briefly, the relative Gibbs energy gradually decreases as the self-assembly proceeds in 1,4-dioxane/mesitylene (Fig. 2a, b, Supplementary Fig. 3–6), indicating this self-assembly process is energetically favorable. The distance between each stacked TAPB ranges from 3.6 to 3.8 Å. However, due to the nonplanar nature of the TAPB structure, there is an offset between adjacent TAPB monomers (Fig. 2a), which may profoundly influence the crystalline structure and properties of the TAPB-TA COF. Solvent has a significant influence on molecular self-assembly^28^. The relative Gibbs energy gradually increased as the TAPB self-assembly proceeded in methanol, suggesting that the self-assembly of TAPB in methanol is energetically unfavorable. This conclusion is consistent with MS measurements that indicate fewer TAPB aggregates are observed in methanol solution.

The aggregation of TAPB monomer was further proved by ultraviolet-visible spectroscopy (UV-Vis), which has been frequently used for characterizing the self-assembly of molecules in solution^29,30^. The UV-Vis spectra of TAPB were measured in TAPB-TA growth solution (1,4-dioxane/mesitylene/water/acetic acid = 1/0.25/0.25/0.375). Only one adsorption peak at around 300 nm was observed when the TAPB concentration was 0.56 mM (Fig. 2f). As the concentration gradually increased to 17.8 mM (the concentration for COF growth), additional adsorption peaks at 556, 657, and 802 nm were detected (Fig. 2g), confirming the concentration-dependent aggregation behavior of the TAPB during COF growth process.

The self-assembly of TAPB explains why TAPB-TA crystalline domains can be formed within such a short time (30 s). When the degree of freedom of individual monomers is high in solution, polymerization of these unrestricted monomers is not different from polymerization to form conventional polymers or porous organic polymers, and only amorphous products are obtained^31,32^. In contrast, the degree of freedom of the TAPB is greatly reduced in the assembled structures. This changes the polymerization directionality and makes it easier for the assembled structures to directly polymerize into a COF structure with crystalline domains. In other words, when TAPB self-assembly is inhibited, it is difficult to form crystalline domains during fast polymerization. This reasoning is supported by the observation that, without detecting obvious TAPB self-assembly, only amorphous TAPB-TA polymers were obtained from methanol growth solution within 15 min (Fig. 1f).

To exclude the possibility that the Schiff-base formation reaction changes with different experimental conditions, which in turn may alter COF crystallinity, we considered the influence of solvent properties and TAPB self-assembly on the reaction mechanism. DFT calculations indicated that the acetic acid-catalyzed Schiff-base reactions follow a proton shuttling mechanism (Fig. 2d). The highest reaction energy barrier between TAPB and TA in 1,4-dioxane/mesitylene was 11.7 kcal/mol, much lower than that of reaction under uncatalyzed conditions (46.6 kcal/mol, Supplementary Fig. 7), confirming the decisive role of acetic acid as a catalyst in Schiff-base formation reactions^33^. When the solvent was changed to methanol, Schiff-base formation reaction proceeded with the same proton shuttle mechanism, and the energy barrier was reduced from 11.7 to 10.8 kcal/mol. As the TAPB stacked together, the reaction between the TAPB stacked structure and TA proceeded by a Schiff-base reaction mechanism identical to the above, and the energetic barrier slightly increased from 11.7 to 12.4 kcal/mol (Fig. 2e). These results indicate that neither solvent property nor TAPB self-assembly dramatically affects Schiff-base formation. Therefore, the significant solvent influence seen on TAPB-TA crystallinity can be attributed to different self-assembly behaviors of TAPB and oligomers in different solvents. However, it should be noted that a considerable amount of amorphous product was observed in the TAPB-TA material synthesized within 30 s, as indicated by the big bulge in the PXRD pattern at a 2θ value of 20° (Fig. 1d). The amorphous phase can be explained by the fast condensation of monomers without self-assembly.

As the TAPB-TA growth time extended from 30 s to 3 h, MS signals from the self-assembled structures of TAPB and oligomers further decreased (Supplementary Fig. 2). At the same time, the yield increased from 32% to 70%, implying that monomers, oligomers, and their assembled structures continuously polymerized into COF products. The decrease in FTIR aldehyde signal intensity (Fig. 1f), the increase in quantitative PXRD [100] reflection signal intensity (Fig. 1b), the narrowing of PXRD peaks (e.g., [100] reflection, Fig. 1c), the appearance of new PXRD peaks (e.g., [210] and [001] reflections, Fig. 1d), the increase in surface area (Fig. 1g), and the more uniform pore size distribution (Fig. 1i) indicate that longer growth time give TAPB-TA COF higher degrees of polymerization, fewer defects, and larger crystalline domains.

As the TAPB-TA growth time further extended from 3 h to 1 day, the PXRD signal intensity of the crystalline phases increased significantly (Fig. 1c). However, the intermediate species in the growth solution, yield, and surface area of the COF products did not change obviously (Fig. 1). These observations suggest that the increased COF crystallinity is not due to nucleation condensation of monomers, because yield did not increase obviously (from 66.7% to 68.2%, Fig. 1g). Therefore, the only possibility for such an increase in crystallinity is that the amorphous phase transforms into the crystalline phase. This implies a growth mechanism different from the frequently proposed nucleation growth mechanism of COFs^18,19,21^. We refer to this COF growth mechanism as self-healing. Based on these new observations, the TAPB-TA growth process can be divided into two stages, nucleation and self-healing (Fig. 1h). In the nucleation growth stage, monomers condense together and form COF products. Crystallinity mainly arises from the monomer nucleation; therefore, this process is heavily influenced by monomer concentration and accompanied by yield increase^17–20^. In the self-healing growth stage, crystallinity increases mainly due to the conversion of the amorphous phase into the crystalline phase. This process causes no apparent change in the yield, and no addition of monomer is needed. We considered using TEM to image the crystalline domain variation during the COF growth process. However, the crystalline domain size was found to vary significantly from spot to spot even for the same COF sample, preventing us from drawing a clear relationship between crystalline domain size and growth time (Supplementary Fig. 8). We also tried to use ^13^C solid-state nuclear magnetic resonance spectroscopy (^13^C SSNMR) to characterize the COF growth process, but only detected limited differences for COFs with different growth time (Supplementary Fig. 9), indicating that the chemical composition of the TAPB-TA COF does not change significantly during the growth process.

### Intermediate tracing study on self-healing growth

**Fig. 3:**
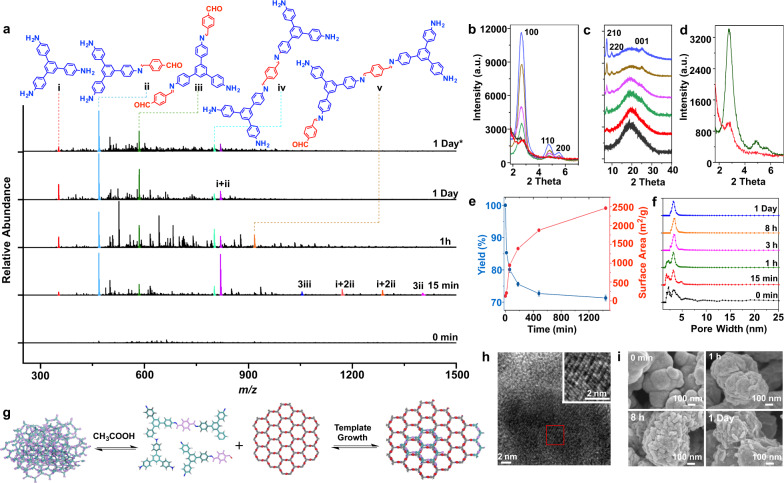
Self-healing growth of TAPB-TA COF. **a** MS characterization of the molecular species in self-healing solution with different reaction time; *mass spectrum from TAPB-TA nucleation growth solution. Quantitative PXRD patterns of TAPB-TA with different self-healing time, 2θ value ranging from (**b**) 1.8° to 7.0°, and (**c**) 7.0° to 40°; growth time: 0 min (black), 15 min (red), 1 h (green), 3 h (purple), 8 h (orange), and 1 day (blue). **d** Quantitative PXRD patterns of TAPB-TA with 15 min growth time (green) and 15 min self-healing time (red). **e** Yield and surface area of TAPB-TA COF versus growth time. **f** Pore size distribution of the TAPB-TA COF with different self-healing time. **g** Proposed TAPB-TA COF self-healing mechanism. **h** TEM image of TAPB. **i** SEM images of TAPB-TA COF with different self-healing time.

While the nucleation growth mechanism has drawn significant attention, the self-healing mechanism has been largely unexplored in COF growth. To further study the self-healing growth mechanism of TAPB-TA by MS, amorphous TAPB-TA polymers were treated under the same conditions as that for nucleation growth of TAPB-TA COF, except that no monomer was added to the self-healing solution. At the beginning of the self-healing process, no monomer or oligomer was detected in the self-healing solution (Fig. 3a, Supplementary Figs. 10 and 11, Supplementary Table 1), indicating that the amorphous TAPB-TA polymer was impurity-free. After 15 min, self-healing intermediates, including TAPB monomer, compound-ii to -iv, and their aggregates, were detected in the self-healing solution (Fig. 3a). At the same time, the yield decreased from 100 to 85.3%, indicating that around 15% of the amorphous TAPB-TA decomposed and dissolved into the self-healing solution. However, the TAPB-TA crystallinity only slightly increased, and remained much lower than the crystallinity of TAPB-TA grown for 15 min via the nucleation mechanism (Fig. 3d), confirming the importance of self-assembly. As the self-healing reaction proceeded to 1 h, the crystallinity of the self-healed TAPB-TA increased obviously, and the crystalline lattice could be observed by TEM (Fig. 3h). When the self-healing time reached 8 h, the yield decreased from 85.3% to 72.7%. Quantitative PXRD data showed that the crystallinity of the self-healed TAPB-TA increased continuously (Fig. 3b). The intensity of the amorphous signal decreased accordingly (Fig. 3c), confirming the transformation of the amorphous phase into the crystalline phase. This observation was consistent with the increased surface area and uniform pore size distribution observed as the self-healing proceeded (Fig. 3e, f). As the self-healing time further extended to 1 day, the yield did not change much, indicating that the decomposition and condensation reactions of the TAPB-TA reached equilibrium.

Based on the above results, we propose that during the self-healing process, the amorphous TAPB-TA partially decomposed into intermediates, such as monomers and oligomers. These intermediates then grew into crystalline domains, realizing the transformation from amorphous to crystalline phase (Fig. 3g). Because no oligomers as large as nanosheets were detected in the self-healing solution, the possibility that the monomers or oligomers polymerized into nanosheets and then stacked together to form crystalline domains can be excluded. In addition, monomers/oligomers may adsorb on the existing TAPB-TA surface, which acts as a template to direct the assembly and pre-arrangement of monomers/oligomers, thereby facilitating the formation of ordered crystalline domains^34,35^. This template growth mechanism can be proved by investigating the morphology of the COF crystals because partial decomposition of the amorphous phases and the formation of new crystalline domains will change the morphology. As shown by the scanning electron microscope (SEM) image in Fig. 3i, the surface of the TAPB-TA COF crystals gradually changed from smooth to very rough as the self-healing proceeded, indicating the occurrence of templated growth. Notably, the templated growth mechanism may have great potential applications. For example, monomers/oligomers in the self-healing solution could grow onto different surfaces (e.g., graphene) present in the solution^36^. Moreover, it should be noted that the nucleation and self-healing growth mechanisms may co-exist in the same system if the COFs are linked by reversible covalent bonds. However, COFs linked by irreversible covalent bonds, such as dioxin-linked COF-316^37,38^, may only form crystalline domains via the nucleation growth mechanism.

### 2D COF single crystal growth and structure resolving

**Fig. 4:**
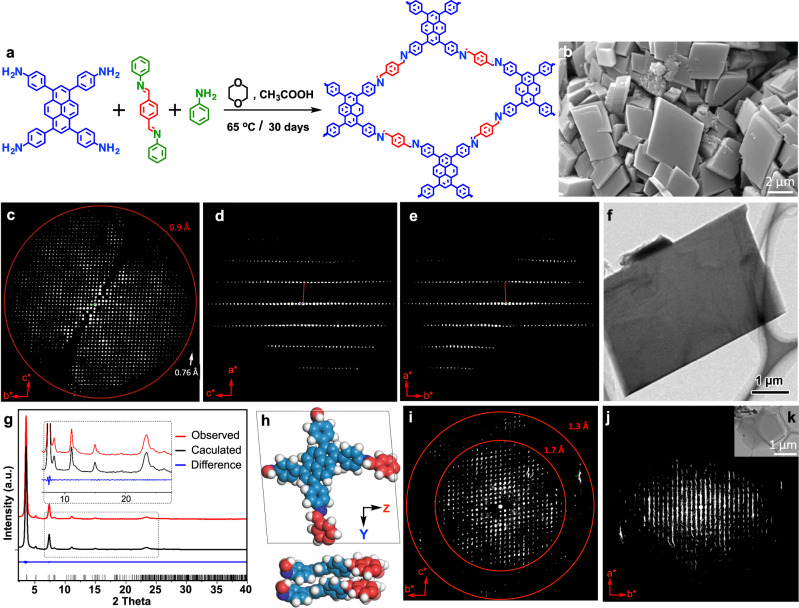
Growth of 2D COF Py-1P single crystals. **a** Chemical structure of Py-1P COF and synthesis from DTA (blue) and PPA (red/green). **b** SEM image of Py-1P crystals. **c**–**e** 3D reciprocal lattice of Py-1P reconstructed from the cRED data viewing along the (**c**) [100], (**d**) [010], and (**e**) [001] directions. **f** TEM image of the Py-1P crystal used for cRED data collection. **g** Pawley fitting of powder X-ray diffraction (λ = 1.54178 Å) for Py-1P. Red line: observed; black line: calculated; blue line: difference; black bars: Bragg conditions. **h** Single crystal structure of Py-1P COF. A reconstructed 3D reciprocal lattice of the Py-1P-6 structure viewing along the (**i**) [100] and (**j**) [001] directions. **k** TEM image of the Py-1P-6 crystal.

The above COF growth mechanism study suggests that to grow 2D COF single crystals, growth conditions should be well-designed according to the COF chemical structure. Specifically, monomers prone to self-assembly and stacking into ordered structures are more likely to grow into single crystals. Additionally, amorphous polymers formed by the condensation of randomly arranged monomers should be avoided. The inhibition of random condensation can be achieved by slowing down the polymerization speed, so that monomers and oligomers can fully self-assemble and pre-arrange before polymerization. The crystal seeds resulting from nucleation condensation will then facilitate single crystal growth via a template growth mechanism. Based on these analyses, we selected 4,4′,4″,4‴-(1,9-dihydropyrene-1,3,6,8-tetrayl)-tetraaniline (DTA) and (1,4-phenylene)bis(*N*-phenylmethanimine) (PPA) monomers as a model example to grow Py-1P Schiff-base COF single crystals (Fig. 4a). DTA is a more promising monomer than TAPB to grow 2D COF single crystals because it incorporates a pyrene group with a large π-conjugated planar structure, giving the monomer a relatively more planar structure with enhanced inter-molecular attractions (e.g., π-π interaction) that facilitates self-assembly and pre-arrangement into ordered structures. This feature should enable high-quality crystal seeds to form during the nucleation growth stage. In contrast, it is difficult to produce suitable crystal seeds with TAPB because of its non-planar structure and slight offset in its stacked assemblies. PPA is an analogue to terephthaladehdye (TA), and was chosen because its aniline-protected aldehyde groups can react with the amine groups of DTA at a much slower speed than the unprotected aldehyde groups in TA. This provides sufficient time for prearrangement of monomers and oligomers to facilitate crystal growth via the template mechanism. Py-1P single crystals were grown in 1,4-dioxane with the catalysis of acetic acid at 65 °C, and excess aniline was added as a modulator to further reduce the polymerization speed^39^. MS measurements confirmed that DTA monomer could indeed self-assemble in the single crystal growth solution (Supplementary Fig. 12, Supplementary Table 2). While TAPB-TA crystals only formed nanocrystalline domains under the tested conditions, micron-sized Py-1P COF single crystals were successfully obtained after 30 days of growth. PXRD (Supplementary Fig. 13) and FTIR (Supplementary Fig. 14) characterizations indicated the successful reaction between the monomers.

Typical Py-1P crystals have a rhombus plate-like morphology with a size of 2-5 μm (Fig. 4b). Due to the thin thickness of the 2D crystals, it is impossible to obtain single-crystal X-ray (SCXRD) analysis. Therefore, continuous rotation electron diffraction (cRED)^40^ was applied for the structural analysis of the Py-1P single crystals. cRED data show that the Py-1P crystal has an impressively high crystallinity, with the highest diffraction observed at a resolution of 0.76 Å (Fig. 4c–f). The 3D reciprocal lattice reconstructed from cRED data shows that Py-1P crystallizes in a triclinic system, with the unit cell parameters of *a* = 3.93 Å, *b* = 23.39 Å, *c* = 23.54 Å, *α* = 84.5°, *β* = 87.1°, and *γ* = 87.1° (Fig. 4c–e, h). These unit cell parameters are different from what have been predicted^41^. The space group *P*1 was used for further analysis. To confirm the unit cell parameters, Pawley fit against powder X-ray diffraction data was applied (Fig. 4g and Supplementary Table 3). Having such high-resolution data, the framework structure of Py-1P was determined by direct methods: among the 60 C and N atoms, 58 were found directly. The two missing C atoms were added according to the molecular structure of the monomers. The details of data collection and refinement are summarized in Supplementary Table 4. The PXRD pattern of Py-1P simulated using the structural model obtained from the single-crystal analysis matched well with the observed pattern (Supplementary Fig. 15). These results confirm the AA stacking structure of 2D COFs that has been widely predicted in the literature^42,43^. However, after analyzing over 200 cases of single crystals, we found that the Py-1P crystals with AA stacking roughly account for only 10% of the crystals encountered in our measurements. The remaining 90% of the crystals adopt a previously unknown stacking structure (Fig. 4i–k). Viewing along the [100] direction, the reciprocal lattice of this previously unknown structure shows similar 2D symmetry and intensity distributions to the AA structure, which indicates a similar 2D building layer (Fig. 4c and i). Interestingly, when viewing along the [001] direction, especially at the high-resolution region (Fig. 4e and j) that this structure has a periodicity of 6 individual layers stacked along the *a*-axis with an interlayer distance of 3.7 Å, and their unique stacking behavior can also be identified from the unit cell parameters as *a* = 22.41 Å, *b* = 24.16 Å, *c* = 24.74 Å, *α* = 82.0°, *β* = 108.3°, and *γ* = 93.6°.

It is interesting to observe that 2D COFs having identical chemical structures can form different crystal structures. We have named the newly discovered crystal form Py-1P-6. The reason that Py-1P-6 can be formed is possibly because of the non-planar nature of the monomer, which leads to interlayer offset during the nucleation and template growth processes. Due to the high complexity of Py-1P-6, its single crystal structure cannot be resolved at present. It is difficult to distinguish Py-1P-6 from AA stacked Py-1P by PXRD; however, its discovery by 3DED may influence the applications of 2D COFs in catalysis, gas sorption, and membranes. The discovery of this abundant, multi-layer structure implies that the majority of actual 2D COF stacking structures may, in reality, exist in a very different form from the widely assumed AA stacking model. Currently, it is challenging to control the Py-1P/Py-1P-6 ratio and separate those crystals, and relevant studies are in progress.

## Discussion

By tracing 2D COF growth intermediates with MS, two different growth mechanisms, namely nucleation and self-healing, were proposed in the present study. Our study indicates that, in order to obtain 2D COFs with high crystallinity, self-assembly and pre-arrangement of monomers and oligomers are important factors, which in turn can be tuned by the chemical structures of monomers and COF growth conditions (e.g., solvent selection). As a proof of the 2D COF growth theory developed above, high quality 2D COF Py-1P single crystals were grown by following the guidelines obtained during the 2D COF growth mechanism study, resulting in successfully resolving of the 2D COF single crystal by cRED for the first time. On the one hand, the resolving of 2D COF single-crystal structure confirmed the widely proposed AA stacking structure, providing a solid cornerstone for our understanding and applications of 2D COFs. On the other hand, the discovery of a previously unknown multiple-layer stacked structure challenges our current knowledge and implies new opportunities for the further studies in 2D COF field.

## Methods

### DFT calculation

All the structures were optimized with the B3LYP-D3BJ/def2-SVP method. Vibrational frequencies were computed at the same level to verify no imaginary frequency in energy minima and only one imaginary frequency in transition states. The transition states were further confirmed by directly connecting the reactants and products through the intrinsic reaction coordinate (IRC) analysis. The single-point energy calculations were conducted using the B3LYP-D3BJ/def2-TZVP method with the polarizable continuum model (PCM) to incorporate the solvent effect. For TAPB stacking and the reaction of TA with single TAPB, the single-point energies were calculated separately with methanol (ε = 32.613) and dioxane (ε = 2.2099)/mesitylene (ε = 2.2650) mixture (4:1 by volume, ε = 2.2209) as the solvent, while for the reaction of TA with single TAPB or stacked TAPB_2_, only dioxane/mesitylene mixture was used as the solvent. All the reported energies are the Gibbs energies (*G*) at 338.15 K. All the calculations were performed with the Gaussian 16 package^44^.

To evaluate the trend of TAPB assembly prior to its reaction with TA, TAPB stacking was firstly studied through DFT calculations. Two, three, four, and five TAPB stacking structures were considered. Further increasing the number of TAPB stacking was not included due to extremely high computational cost. Supplementary Fig. 3 illustrates the fully optimized structures of TAPB_n_ (n = 2–5) complexes.

### Transmission electron microscopic (TEM) analyses

Samples for transmission electron microscopy observation were taken from the solution after synthesis. A droplet of the solution containing Py-1P single-crystals was transferred onto a carbon-coated copper grid. The observation was performed on a JEOL JEM2100 microscope and operated at 200 kV (Cs 1.0 mm, point resolution 0.23 nm). Images were recorded with a Gatan Orius 833 CCD camera (resolution: 2048 × 2048 pixels; pixel size: 7.4 µm). Electron diffraction patterns were recorded with a Timepix pixel detector QTPX-262k (512 × 512 pixels, pixel size 55 µm, Amsterdam Sci. Ins.). All the experiments were conducted under low-dose conditions.

### Continuous rotation electron diffraction (cRED) collection

The data were collected using the software *Instamatic*^40^. A single-tilt tomography holder was used for the data collection, which could tilt from −70° to +70° in the TEM. The aperture used for cRED data collection covers an area of about 1.0 μm in diameter. The speed of goniometer rotation is 0.45° s^−1^, and the exposure time is 0.5 s per frame. Data were collected in 3.1 min to minimize the beam damage and to maximize the data quality. One frame in every 20 frames was used to generate image to trace the crystal. The covered tilt angle is 83.5°. The ED data were processed using XDS package^45^. The dataset has a high signal-to-noise ratio within the resolution of 0.90 Å. Due to the preferred orientation of the 2D crystal and the limitation of the goniometer tilting range, the data completeness is 41.8%. The *R*_*int*_ value is 0.099. The cRED data have sufficient quality to determine the framework structure of Py-1P by direct methods using the program SHELX-2014^46^. Among the 60 C and N atoms, 58 were found directly. The two missing C atoms were added according to the molecular structure of the monomers. The final refinement was done using SHELXL-2014, and data converged to *R*_*1*_ = 0.135, using 4003 reflections and 185 parameters. The details of data collection and refinement were summarized in Supplementary Tables 3 and 4. PXRD pattern of Py-1P was simulated using the structural model obtained from the single-crystal analysis, and it matches well with the observed pattern (Supplementary Fig. 15).

### Growth of Py-1P COF single crystals

*Solution-1*: terephthalaldehyde (5.4 mg, 0.04 mmol) was dissolved in 1,4-dioxane (0.3 mL), to which aniline (41 μL, 0.44 mmol) was added to form a clear solution; then aqueous acetic acid solution (90 μL, 6 M) was added to the mixture. Flake crystals of (1,4-phenylene)bis(*N*-phenylmethanimine) gradually formed, and the mixture was kept at room temperature for 15 min. *Solution-2*: 1,3,6,8-tetrakis(4-aminophenyl) pyrene (10.0 mg, 0.018 mmol) was dissolved in 1,4-dioxane (0.5 mL) under sonication. The suspension was placed at 65 °C to obtain a clear brown solution. Then, the heated *solution-2* was added to *solution-1*, and the obtained clear brown solution was placed at 65 °C for 1 to 3 months.

## Supplementary information

Supplementary Information

## Data Availability

All data are available in the main text or the supplementary information. The crystallographic data for single crystal Py-1P has been deposited at the Cambridge Crystallographic Data Centre (CCDC, free of charge at https://www.ccdc.cam.ac.uk) under the deposition number CCDC 2090060.
