# Supplementary material for: Growing single crystals of two-dimensional covalent organic frameworks enabled by intermediate tracing study
Source: Nat Commun. 2022 Mar 16;13:1370. doi: 10.1038/s41467-022-29086-x (PMC8927472; doi:10.1038/s41467-022-29086-x)
Supplement: Supplementary file 1 — Supplementary Information [file 41467_2022_29086_MOESM1_ESM.pdf]

Supplementary Information

Growing Single Crystals of Two-Dimensional Covalent Organic Frameworks Enabled by Intermediate Tracing Study

Chengjun Kang, Kuiwei Yang, Zhaoqiang Zhang, Adam K. Usadi, David C. Calabro, Lisa Saunders Baugh, Yuxiang Wang, Jianwen Jiang, Xiaodong Zou, Zhehao Huang,* Dan Zhao*

*E-mail: zhehao.huang@mmk.su.se, chezhao@nus.edu.sg

Experimental Details

1. Materials and Methods

1.1 Materials

1,3,5,-Tris(4-aminophenyl)benzene (> 93%), terephthalaldehyde (> 98%), 1,4-dioxane (> 99%), 1,3,6,8-tetrabromopyrene (> 98%), 4-(4,4,5,5,-tetramethyl-1,3,2-dioxaborolan-2-yl)aniline (> 98%), and tetrakis(triphenylphosphine)palladium (0) (> 97%) were obtained from Tokyo Chemical Industry CO., LTD (Singapore). Other reagents and solvents were used as received.

1.2 Methods

Instruments and methods. Powder X-ray diffraction (PXRD) patterns were obtained on a Bruker D8 Advance X-ray powder diffractometer equipped with a Cu sealed tube (λ = 1.54178 Å) at a scan rate of 2° min^{-1}. Before PXRD measurements, each sample was prepared by pressing 15 mg of COF powders with 5 kN force into a round flake having 1 cm in diameter. Fourier-transform infrared spectroscopy (FTIR) was performed with a Bio-Rad FTS-3500 ARX FTIR spectrometer. Nuclear magnetic resonance spectroscopy (NMR) was conducted on a Bruker Avance 400 MHz NMR spectrometer (DRX400), with chemical shifts being quoted in parts-per-million (ppm) relative to tetramethylsilane. Field emission scanning electron microscopy (FESEM) images were taken on a JEOL JSM-7610F SEM. Samples were coated with Pt sputtering for 120 s before imaging. Mass spectroscopy (MS) was measured by a Bruker microTOF-Q system in positive ion mode. Here are the detailed conditions for the MS testing: source temperature = 120 °C; dry gas flow rate = 8 L/min; nebulizer pressure = 3 bar; capillary voltage = 3.5 kV; sample injection rate = 3 μL/min. The testing solution was directly injected without further purification unless specified. Brunauer-Emmett-Teller (BET) surface area measurements were performed at 77 K on a Micromeritics ASAP 2020 instrument equipped with commercial software for data calculation and analysis. Before each measurement, the sample (50–80 mg) was degassed at 373 K for 12 h. The N_2 isotherms were collected with a pressure range of 0–0.95 bar.

Synthesis of 1,3,6,8-tetrakis(4-aminophenyl)pyrene[1]

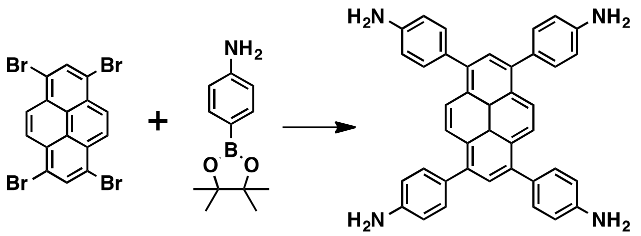

1,3,6,8-Tetrabromopyrene (0.74 g, 1.4 mmol, 1.0 eq.), 4-aminophenylboronic acid pinacol ester (1.5 g, 6.9 mmol, 4.8 eq.), K_2CO_3 (1.1 g, 7.9 mmol, 5.5 eq.), and $Pd(PPh_3)_4$ (165 mg, 0.14 mmol, 10 mol%) were added to a flask under argon atmosphere. Then, degassed 1,4-dioxane (16 mL) and water (4 mL) were added, and the mixture was refluxed (115 °C) for 3 days. After cooling to room temperature, a large excess of water was added to the mixture. The resulting light yellowish precipitate was collected via filtration and washed with

water and methanol. The crude product was purified by dissolving in hot 1,4-dioxane and precipitating in water, filtrating, and drying under a high vacuum. Bright yellow powder was obtained (yield: 78%).

1H NMR (400 MHz, DMSO-d6), δ: 8.13 (4 H), 7.79 (2 H), 7.34 (8 H), 6.77 (8 H), 5.30 (8 H). ^{13}C NMR (DMSO-d6) and high-resolution mass spectroscopy (HRMS) in methanol spectra are shown as follows:

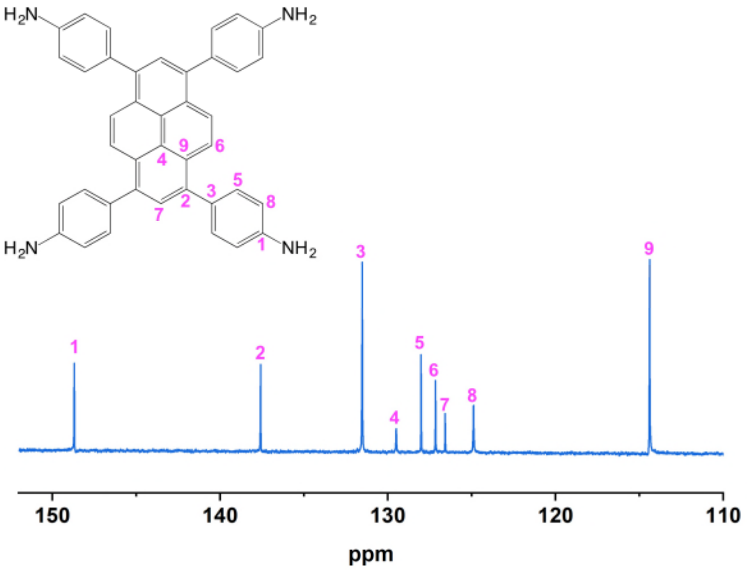

13C NMR of 1,3,6,8-tetrakis(4-aminophenyl)pyrene

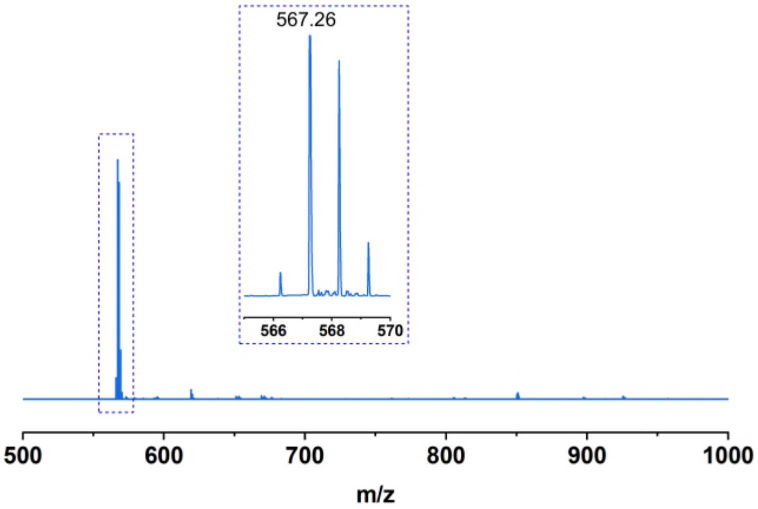

HRMS of 1,3,6,8-tetrakis(4-aminophenyl)pyrene

Growth of TAPB-TA COF by "nucleation" mechanism[2]. 1,3,5-Tris(4-aminophenyl)benzene (55 mg, 0.16 mmol) and terephthalaldehyde (31 mg, 0.23 mmol) were dissolved in 1,4-dioxane (4.8 mL) and mesitylene (1.2 mL), then distilled water (1.2 mL) and acetic acid (1.8 mL) were added to afford a clear solution. The resultant homogeneous solution was sealed and heated at 65 °C for different time. Then, yellow solid powders and the growth solution were separated by filtration. During this process, drying of the COF powders should be avoided. The clear growth solution was subjected to the MS measurement immediately. The solid product was washed with ethanol, immersed in tetrahydrofuran (THF) for 3 days, then further washed by hexane and immersed in anhydrous hexane for 1 day. After being dried under a vacuum, the final COF was obtained.

Growth of TAPB-TA COF by "self-healing" mechanism. Amorphous TAPB-TA polymer (30.0 mg) was added to a mixture of 1,4-dioxane (1.6 mL), mesitylene (0.4 mL), distilled water (0.4 mL), and acetic acid

(0.6 mL). The resultant suspension was sealed and heated at 65 °C for different time. Then, solid powders and the growth solution were separated by filtration. During this process, drying of the solid powders should be avoided. The clear self-healing solution was subjected to the MS measurement immediately. The solid product was washed with ethanol, immersed in THF for 3 days, then further washed by hexane and immersed in anhydrous hexane for 1 day. After being dried under a vacuum, the final self-healed COF was obtained.

2. Additional Figures

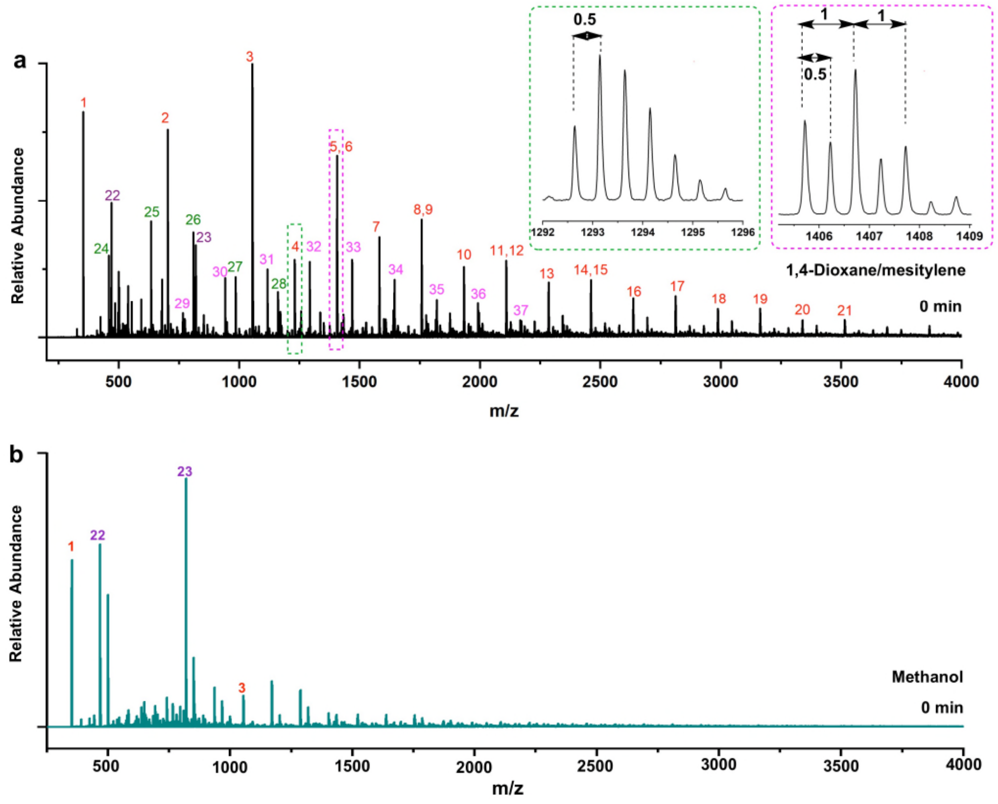

Supplementary Figure 1. MS characterization of TAPB-TA growth. MS spectra of TAPB-TA growth solution with 1,4-dioxane/mesitylene (a) and methanol (b) as the solvent. The unlabeled peaks are fragments of intermediates or intermediates with solvent molecules.

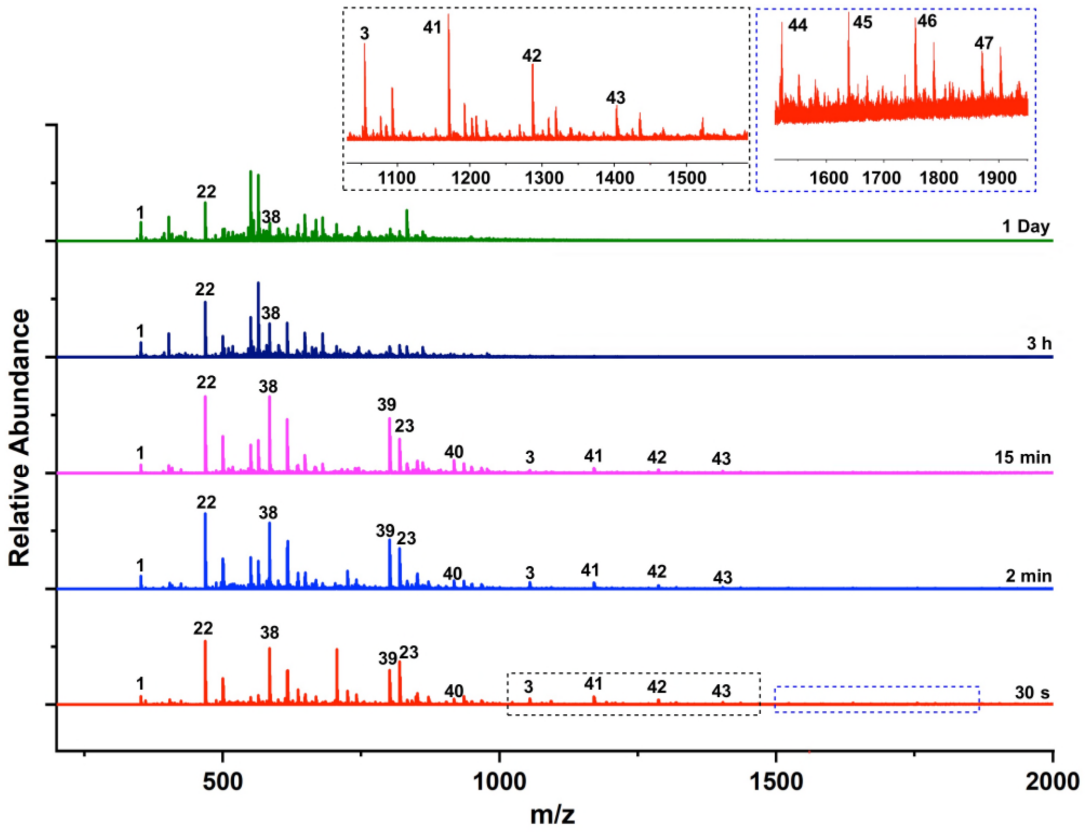

Supplementary Figure 2. MS spectra of TAPB-TA COF growth solution with different growth time (1,4-dioxane/mesitylene was used as the solvent). The unlabeled peaks are fragments of intermediates or intermediates with solvent molecules.

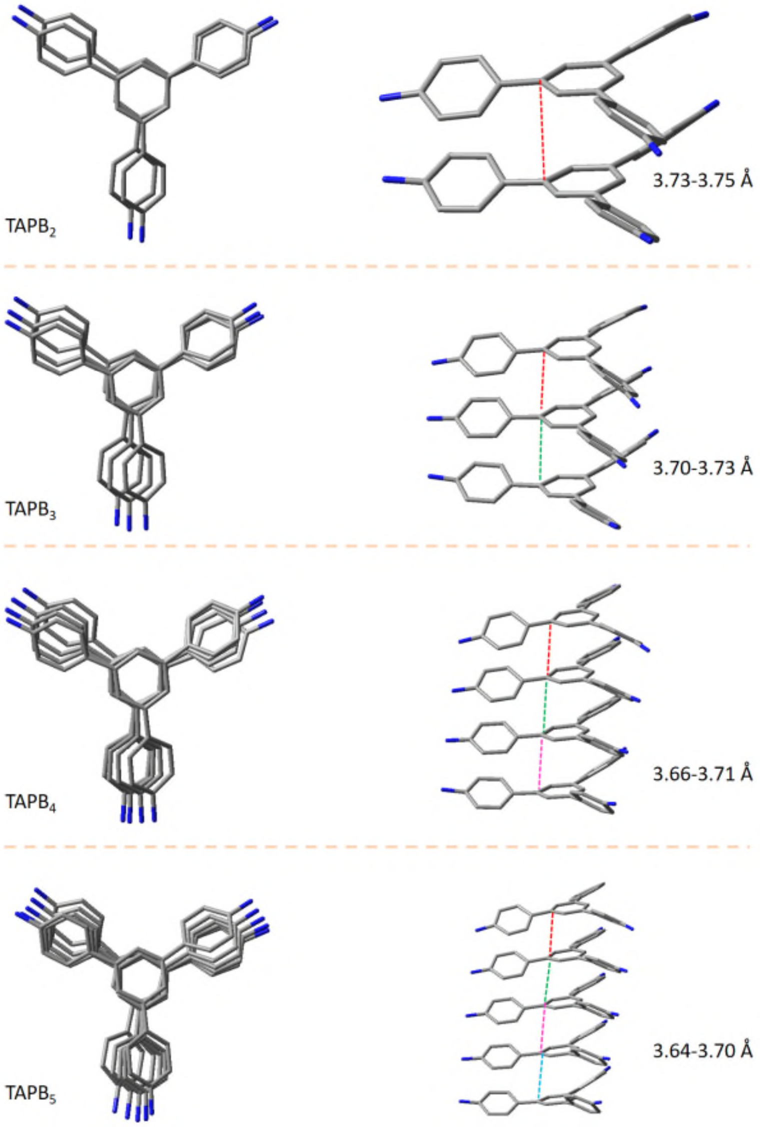

Supplementary Figure 3. Calculated TAPB monomer stacking structures. Top and side views of fully optimized $TAPB_2$, $TAPB_3$, $TAPB_4$, and $TAPB_5$. The H atoms are omitted for clarity. The distances between two C atoms in the central benzene rings of adjacent layers are highlighted in dash lines and labeled aside.

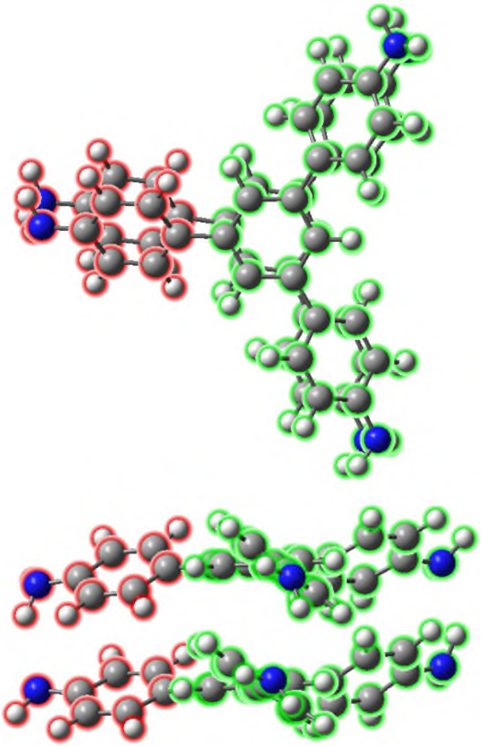

Supplementary Figure 4. Fixation of part of fully optimized $TAPB_2$ in the mechanism study for TA reacting with stacked $TAPB_2$. The fixed parts are indicated in green, while the relaxed parts in red.

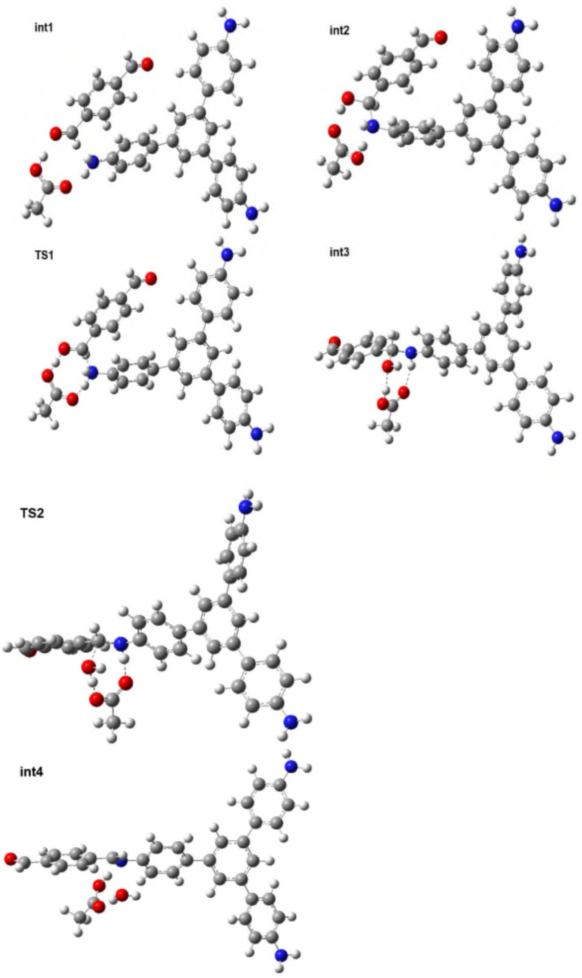

Supplementary Figure 5. Optimized structures involved in TA reacting with single TAPB catalyzed by CH_3COOH.

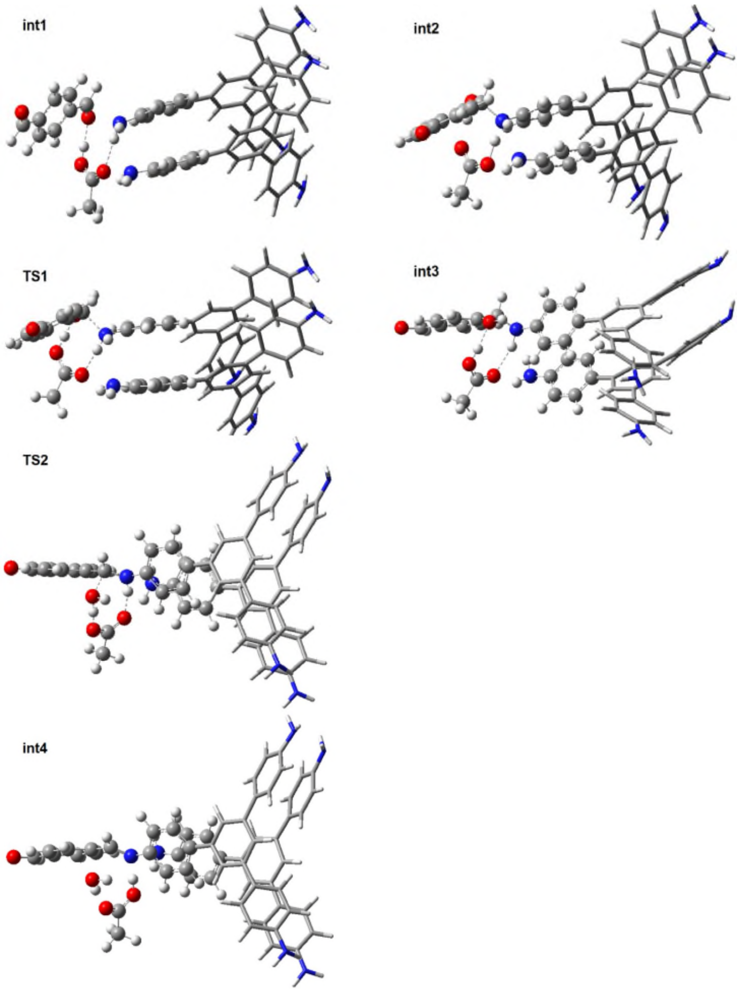

Supplementary Figure 6. Optimized structures involved in TA reacting with stacked $TAPB_2$ catalyzed by CH_3COOH. The fixed parts are indicated as tubes, while the relaxed parts as ball-sticks.

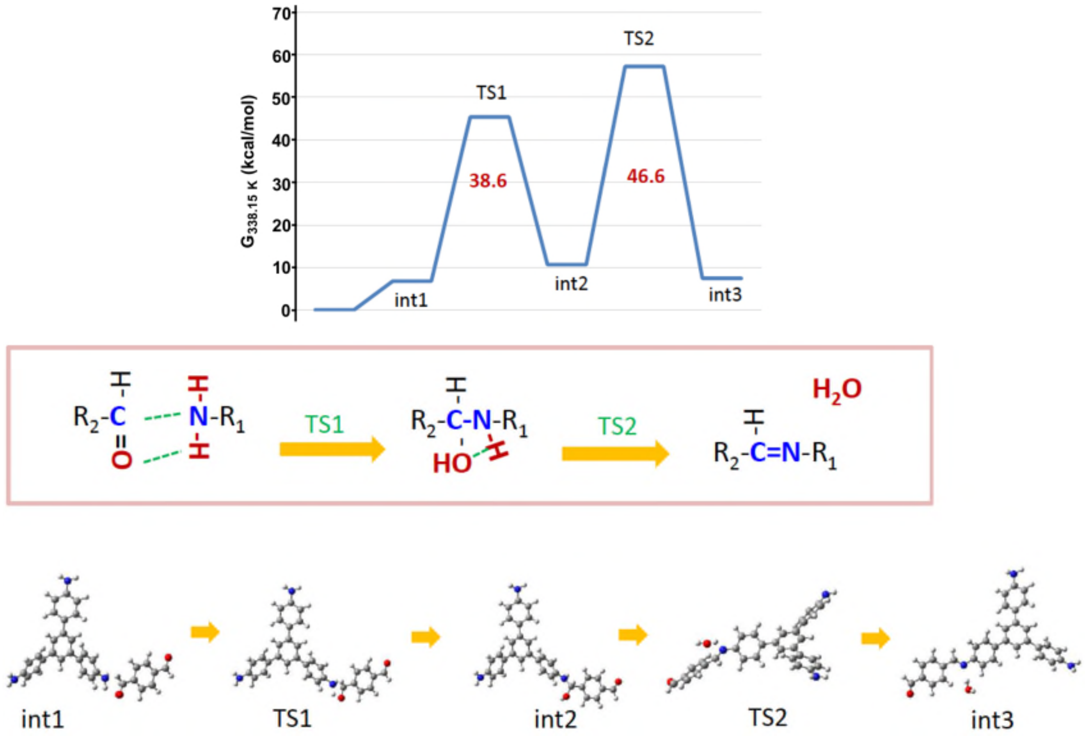

Supplementary Figure 7. Gibbs energy calculation of the Schiff-base reaction between TA and TAPB without catalysis.

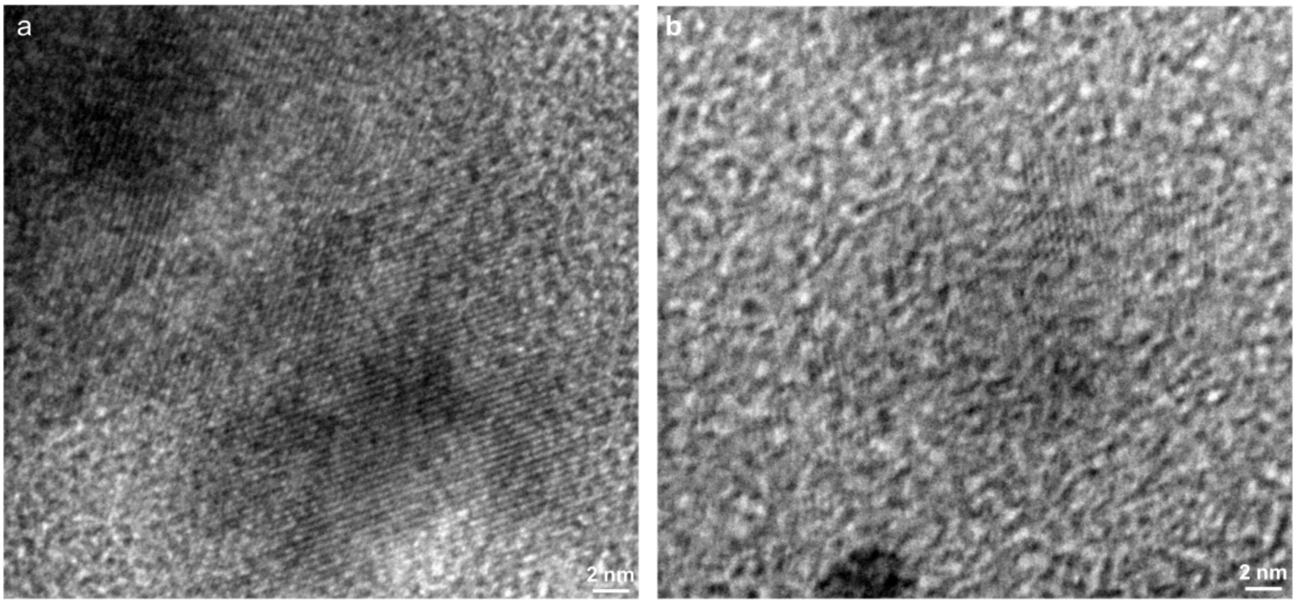

Supplementary Figure 8. TEM images of TAPB-TA COF (1-day growth) at different imaging spots. (a) Spot with large crystalline domains. (b) Spot with limited crystalline domain size.

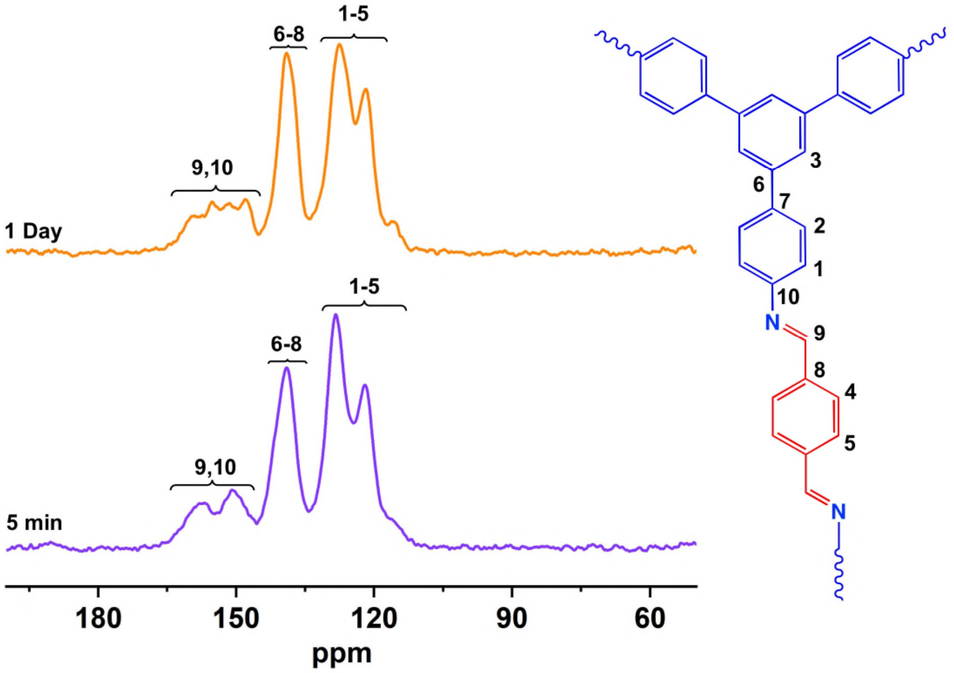

Supplementary Figure 9. ^{13}**C SSNMR spectra of TAPB-TA COF with different growth time.**

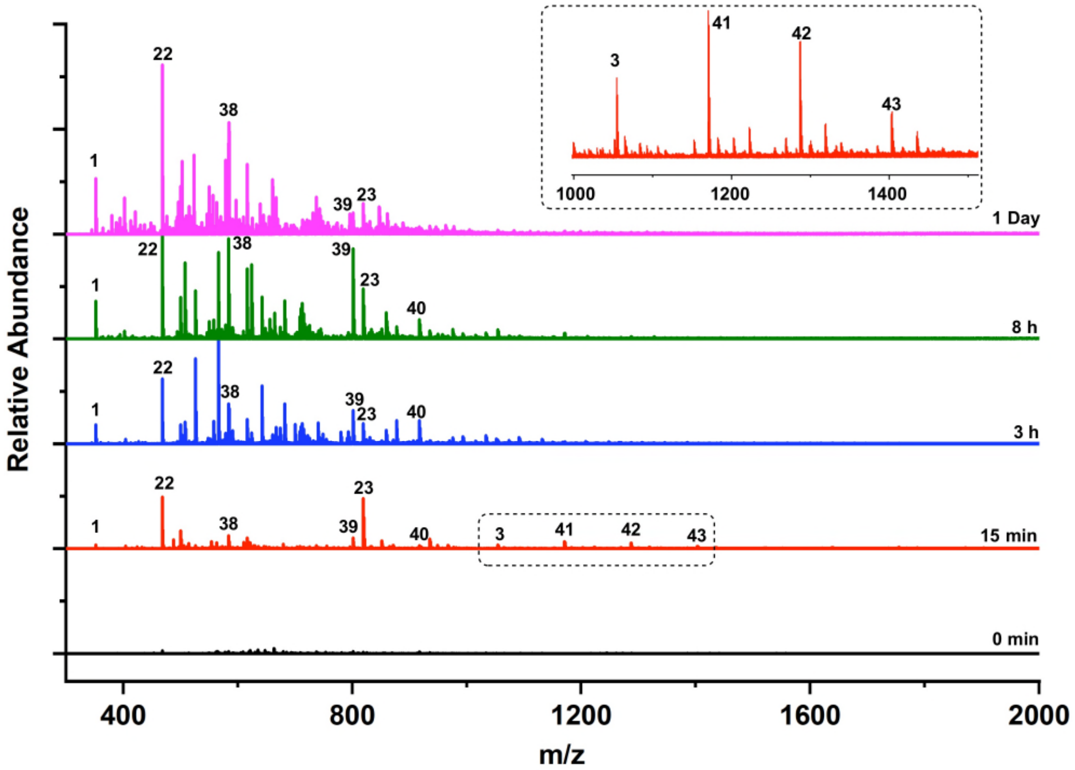

Supplementary Figure 10. MS spectra of TAPB-TA amorphous polymer self-healing solution with different reaction time. The unlabeled peaks are fragments of intermediates or intermediates with solvent molecules.

Supplementary Table 1. Details of MS signals observed on the spectra of TAPB-TA COF growth and self-healing solutions.

Peak number*	Charge	Measured m/z	Calculated m/z	Proposed molecular structure
1	+1	352.18	352.17	i+ H^+
2	+1	703.36	703.34	2i+H^+
3	+1	1054.52	1054.51	3i+H^+
4	+2	1230.11	1230.09	7i+ 2H^+
5	+1	1405.71	1405.68	4i+ H^+
6	+2	1405.71	1405.68	8i+2H^+
7	+2	1581.30	1581.27	9i+2H^+
8	+1	1756.86	1756.85	5i+ H^+
9	+2	1756.86	1756.85	10i+2H^+
10	+2	1933.00	1932.93	11i+2H^+
11	+1	2108.01	2108.02	6i+ H^+
12	+2	2108.01	2108.02	12i+ 2H^+
13	+2	2283.62	2283.60	13i+ 2H^+
14	+1	2459.17	2459.19	7i+ H^+
15	+2	2459.17	2459.19	14i+ 2H^+
16	+2	2634.77	2634.76	15i+ 2H^+
17	+2	2810.38	2810.36	16i+ 2H^+
18	+2	2986.03	2985.95	17i+ 2H^+
19	+2	3161.56	3161.53	18i+ 2H^+
20	+2	3337.11	3337.12	19i+ 2H^+
21	+2	3512.70	3512.70	20i+ 2H^+
22	+1	468.24	468.20	ii+ H^+
23	+1	819.39	819.37	i+ ii+ H^+
24	+2	458.22	458.20	i+ ii+CH_3COOH+2H_3O^+
25	+2	633.81	633.81	2i+ ii+CH_3COOH+2H_3O^+
26	+2	809.41	809.40	3i+ ii+CH_3COOH+2H_3O^+
27	+2	985.00	984.98	4i+ ii+CH_3COOH+2H_3O^+
28	+2	1161.58	1160.57	5i+ ii+CH_3COOH+2H_3O^+
29	+2	766.38	766.39	4i+1,4-dioxane+K^++H^+
30	+2	941.96	941.96	5i+1,4-dioxane+K^++H^+
31	+2	1117.55	1117.54	6i+1,4-dioxane+K^++H^+
32	+2	1293.14	1293.13	7i+1,4-dioxane+K^++H^+
33	+2	1468.73	1468.72	8i+1,4-dioxane+K^++H^+

34	+2	1644.32	1644.30	9i+1,4-dioxane+K^++H^+
35	+2	1819.91	1819.88	10i+1,4-dioxane+K^++H^+
36	+2	1995.43	1995.47	11i+1,4-dioxane+K^++H^+
37	+2	2171.01	2171.05	12i+1,4-dioxane+K^++H^+
38	+1	584.23	584.23	iii+H^+
39	+1	801.36	801.36	iv+H^+
40	+1	917.37	917.39	v+H^+
41	+1	1170.53	1170.54	2i+ii+H^+
42	+1	1286.55	1286.57	i+2ii+H^+
43	+1	1402.59	1402.6	3ii+H^+
44	+1	1521.70	1521.71	3i+ii+H^+
45	+1	1637.72	1636.74	2i+2ii+H^+
46	+1	1753.74	1753.77	i+3ii+H^+
47	+1	1869.81	1869.80	4ii+H^+

*Each peak number corresponds to the peak number labeled in the MS spectra (Supplementary Figures 1, 2, and 10)

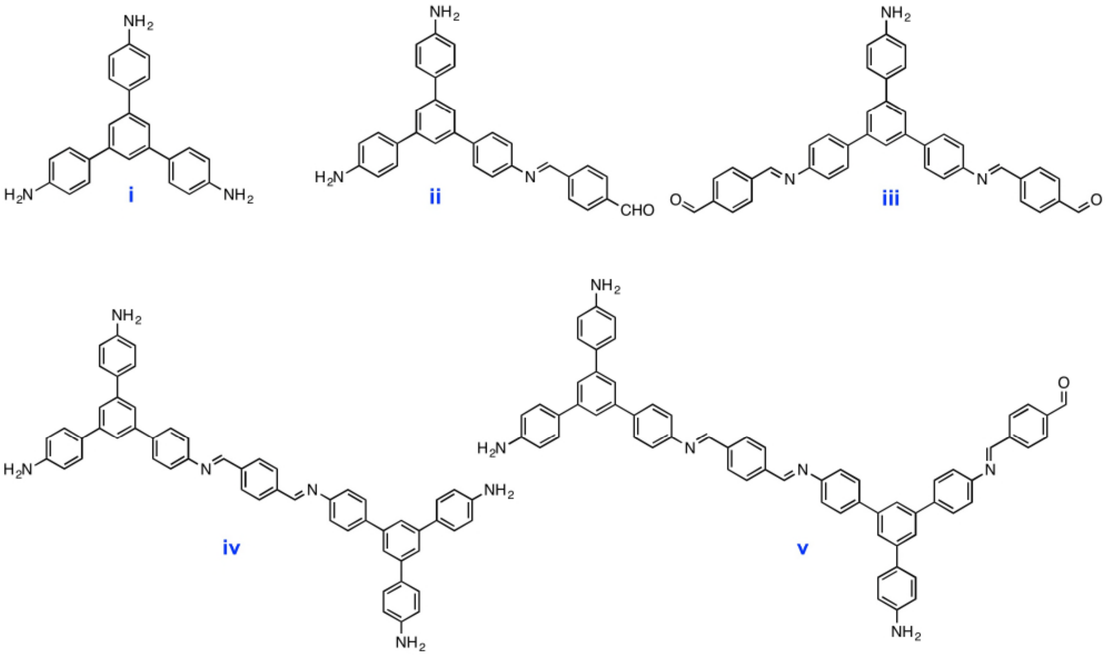

Supplementary Figure 11. Summary of intermediates detected in the nucleation growth and self-healing solutions.

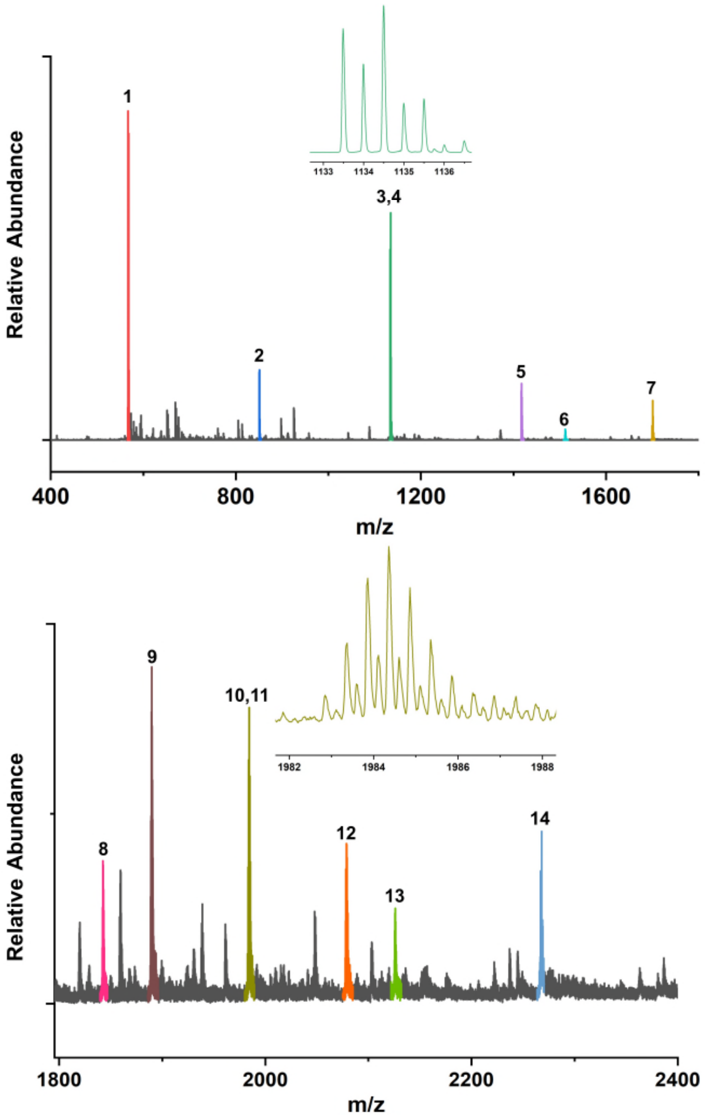

Supplementary Figure 12. MS spectrum of DTA monomer in Py-1P single crystal growth solution with a concentration of 0.5 mg/mL.

Supplementary Table 2. Details of MS signals observed in the spectra of DTA monomer.

Peak number*	Charge	Measured m/z	Calculated m/z	Proposed molecular structure
1	+1	567.26	567.25	1 × DTA+H^+
2	+2	850.37	850.37	3 × DTA+2H^+
3	+1	1333.49	1333.50	2 × DTA+1H^+
4	+2	1333.49	1333.50	4 × DTA+2H^+
5	+2	1416.62	1416.62	5 × DTA+2H^+
6	+3	1510.99	1511.00	8 × DTA+2H^+
7	+1	1699.75	1699.75	3 × DTA+1H^+
8	+4	1841.31	1841.31	13 × DTA+4H^+
9	+3	1888.51	1888.50	10 × DTA+3H^+
10	+2	1982.82	1982.88	7 × DTA+2H^+
11	+4	1982.82	1982.88	14 × DTA+4H^+
12	+3	2077.24	2077.25	11 × DTA+3H^+
13	+4	2124.25	2124.25	15 × DTA+4H^+
14	+1	2265.98	2266.00	4 × DTA+2H^+

*Each peak number corresponds to the peak number labeled in the MS spectra (Supplementary Figure 12)

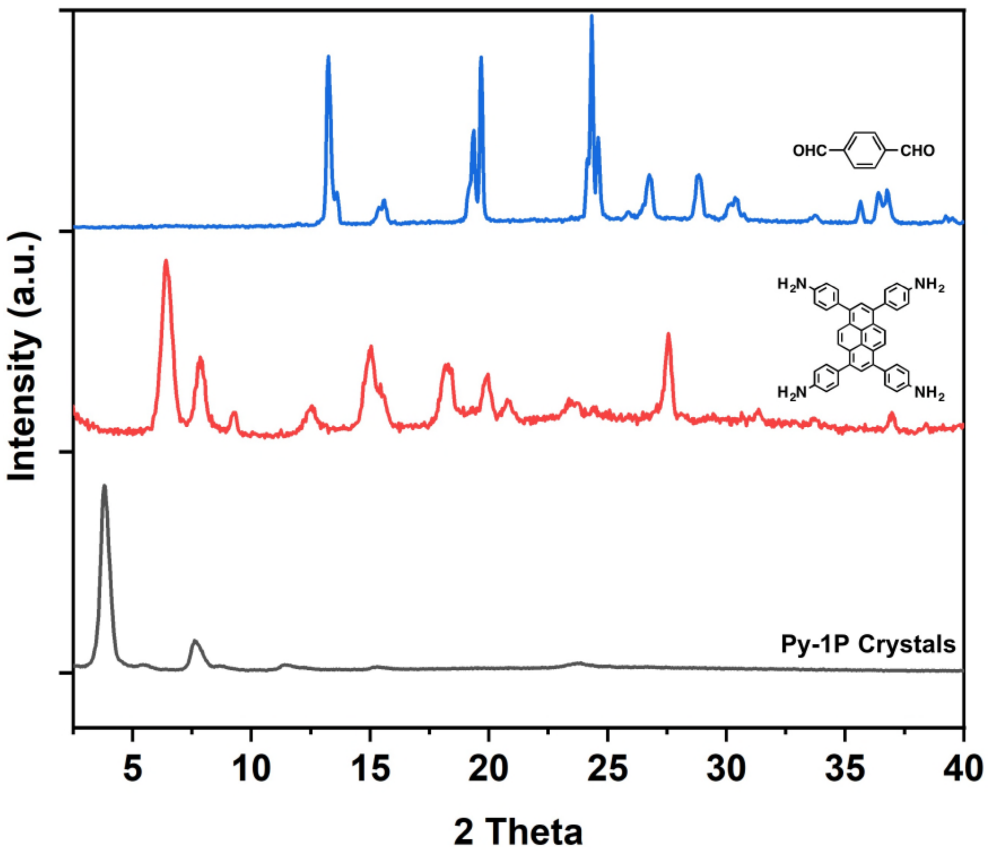

Supplementary Figure 13. PXRD characterization of Py-1P crystals. PXRD patterns of the Py-1P COF single crystal (black), 4,4′,4″,4‴-(1,9-dihydropyrene-1,3,6,8-tetrayl)-tetraaniline (DTA) monomer (red), and terephthalaldehyde monomer (blue).

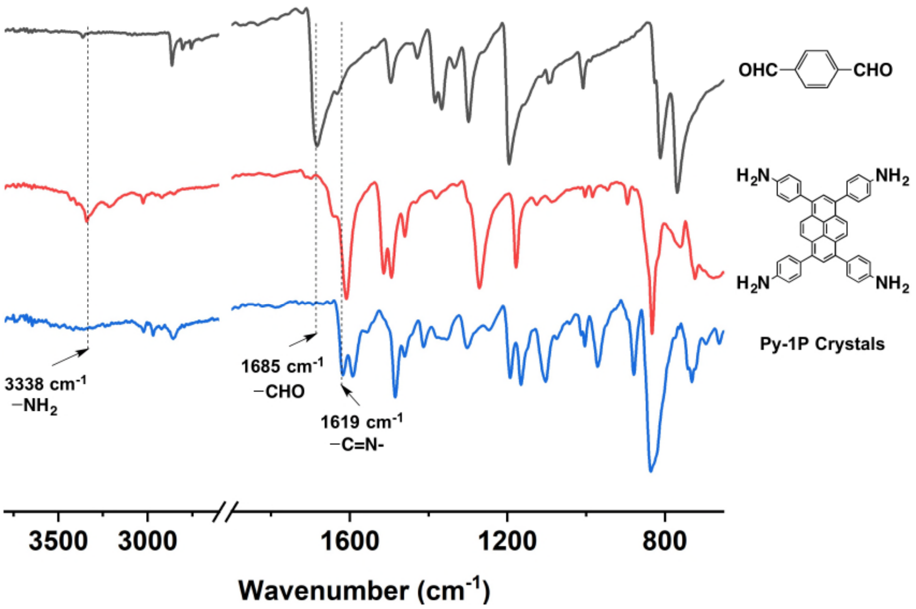

Supplementary Figure 14. FTIR characterization of Py-1P crystals. FTIR spectra of the Py-1P COF single crystal (blue), 4,4′,4″,4‴-(1,9-dihydropyrene-1,3,6,8-tetrayl)-tetraaniline (DTA) monomer (red), and terephthalaldehyde monomer (black).

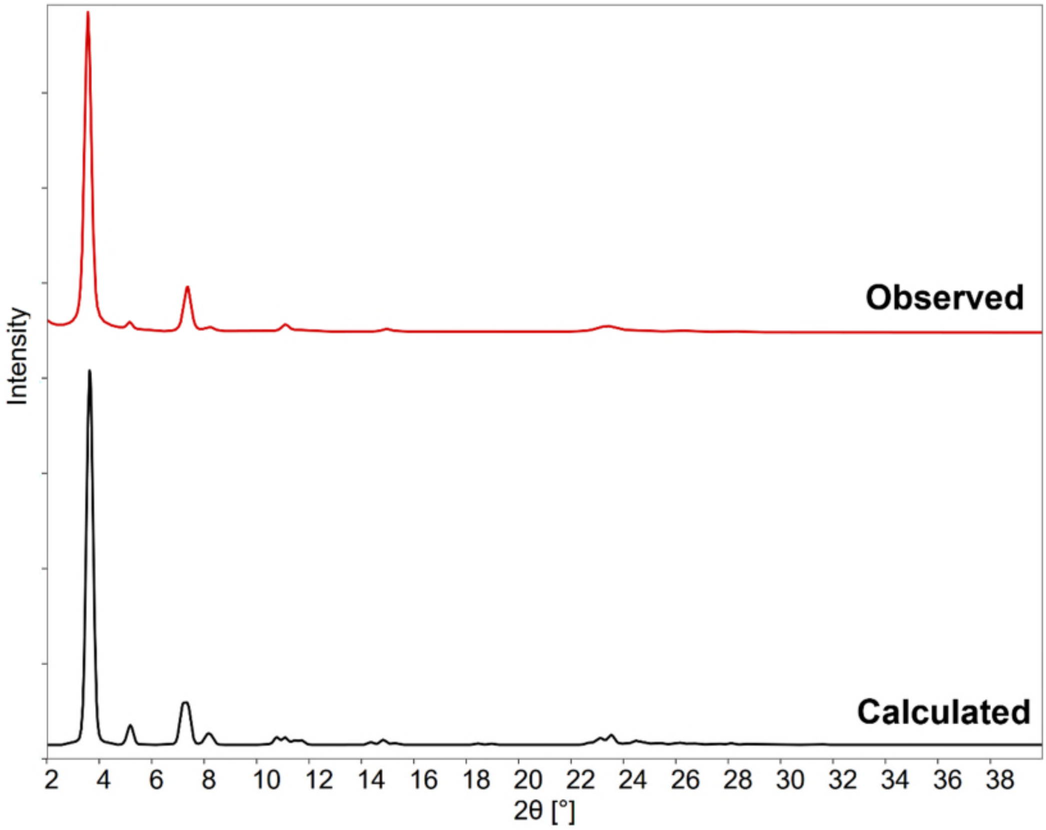

Supplementary Figure 15. Simulated PXRD pattern (λ = 1. 54178 Å) of Py-1P by using the structural model obtained from the single-crystal analysis. It agrees well with the observed PXRD pattern. However, due to peak overlap and similarity of the building layers, it is difficult to distinguish the new phase with different stacking behavior from the PXRD pattern.

Supplementary Table 3. Crystallographic data and Pawley fit results of Py-1P*.

Chemical formula	$C_{56}N_4$
Formula weight	728.60
Crystal system	Triclinic
Space group	*P*1
a (Å)	4.0(3)
b (Å)	24.5(1)
c (Å)	23.9(1)
α (°)	88.7(5)
β (°)	87(4)
γ (°)	89(9)
Temperature/K	298
Wavelength/Å	1.54056
2*θ* range/°	2.29 – 40.01
R_p	0.0119
R_{wp}	0.0165
R_{exp}	0.0159
GOF	1.042

*The large standard uncertainties of the unit cell parameters are due to the severe peak overlap in the PXRD pattern as well as the existence of a closely related but differently stacked phase.

Supplementary Table 4. Single crystal data collection and crystallographic data for Py-1P (λ = 0.0251 Å).

Tilt range (°)	-35.6 to 47.9
Tilt rate ($^{o}\ s^{-1}$)	0.45
Exposure time (s)	0.5
Total number of images	326
Total data collection time (min)	3.1
Beam current	< 0.01 pA
Chemical formula	$C_{56}N_4$
Space group	*P*1 (No. 1)
a (Å)	3.9280(8)
b (Å)	23.393(5)
c (Å)	23.544(5)
α (°)	84.51(3)
β (°)	87.07(3)
γ (°)	87.05(3)
Z	1
Completeness	0.418
Resolution (Å)	0.90
No. of reflections (all unique)	4003
No. of reflections ($F_o>4\sigma(F_o)$)	2176
Refined parameters	185
R_{int}	0.099
R_1 ($F_o>4\sigma(F_o)$)	0.135
R_1 (all reflections)	0.175
Goof	1.034

References

1. Auras, F. *et al.* Synchronized Offset Stacking: A Concept for Growing Large-Domain and Highly Crystalline 2D Covalent Organic Frameworks. *J. Am. Chem. Soc.* **138**, 16703–16710 (2016).
2. Kang, C. *et al.* Interlayer Shifting in Two-Dimensional Covalent Organic Frameworks. *J. Am. Chem. Soc.* **142**, 12995–13002 (2020).
